# HER-3 targeting alters the dimerization pattern of ErbB protein family members in breast carcinomas

**DOI:** 10.18632/oncotarget.6762

**Published:** 2015-12-26

**Authors:** Michalis V. Karamouzis, Georgia Dalagiorgou, Urania Georgopoulou, Afroditi Nonni, Michalis Kontos, Athanasios G. Papavassiliou

**Affiliations:** ^1^ Molecular Oncology Unit, Department of Biological Chemistry, Medical School, National and Kapodistrian University of Athens, 11527 Athens, Greece; ^2^ Laboratory of Molecular Virology, Hellenic Pasteur Institute, 11521 Athens, Greece; ^3^ First Department of Pathology, Medical School, National and Kapodistrian University of Athens, 11527 Athens, Greece; ^4^ Department of Propaedeutic Surgery, Medical School, National and Kapodistrian University of Athens, ‘Laikon’ General Hospital, 11527 Athens, Greece

**Keywords:** HER-3, ErbB, dimerization pattern, proximity ligation assay, breast cancer

## Abstract

Breast carcinogenesis is a multi-step process in which membrane receptor tyrosine kinases are crucial participants. Lots of research has been done on epidermal growth factor receptor (EGFR) and HER-2 with important clinical results. However, breast cancer patients present intrinsic or acquired resistance to available HER-2-directed therapies, mainly due to HER-3. Using new techniques, such as proximity ligation assay, herein we evaluate the dimerization pattern of HER-3 and the importance of context-dependent dimer formation between HER-3 and other HER protein family members. Additionally, we show that the efficacy of novel HER-3 targeting agents can be better predicted in certain breast cancer patient sub-groups based on the dimerization pattern of HER protein family members. Moreover, this model was also evaluated and reproduced in human paraffin-embedded breast cancer tissues.

## INTRODUCTION

The ErbB/HER receptors are type I growth factor receptors with tyrosine kinase (TK) activity; epidermal growth factor receptor 1 (also called EGFR, ErbB1 or HER-1), HER-2 (ErbB2), HER-3 (ErbB3) and HER-4 (ErbB4) [1]. They consist of a trans-membrane hydrophobic domain that separates an extracellular ligand-binding domain and an intracellular TK domain. In order to activate their TK activity, ligands that contain an epidermal growth factor (EGF)-like domain bind to HER receptors. Different EGF-like ligands activate different receptors of the HER family, except HER-2 which has no identified ligand yet [2]. Then, dimer formation between the four receptors occurs to activate the TK domain. Upon activation, HER receptors stimulate downstream pathways that are involved in apoptosis, proliferation, differentiation, angiogenesis, epithelial–mesenchymal transition (EMT) and cell motility [3, 4]. Deregulation and subsequent aberrant signaling of HER protein family due to mutation, amplification or presence of autocrine / paracrine loops enhances the development of breast carcinomas.

Expression of all four HER receptors is necessary during normal development of mammary gland [5]. Expression of HER receptors in breast carcinomas is a common event. HER-2 over-expression is present in 20-30% of breast carcinomas and is associated with dismal prognosis [6]. Additionally, a sub-population of HER-2 over-expressing breast cancer patients expresses a truncated active form, p95 HER-2, which lacks the extracellular domain. Cells carrying the truncated form of HER-2 protein are more prone to constant HER-2 homodimer activity and uncontrolled growth, division and avoidance of apoptosis [7]. HER-3 is often over-expressed in human breast cancer cells due to higher protein expression or increased half-life of the receptor. HER-3 increased expression in breast tumors has been correlated with poor prognostic features, such as increased metastatic potential, high tumor grade and increased recurrent rate [8, 9]. However, it should be noted that a recent meta-analysis did not find any correlation between HER-3 expression and breast cancer survival [10]. Although not frequently over-expressed in breast cancers, HER-4 is correlated with good prognosis and seems to antagonize HER-2-related dismal clinical outcome [11]. Furthermore, over-expression of both HER-3 and HER-4 has been also associated with favorable clinical outcome [12].

There are multiple potential ligands for HER protein family receptors. The ligands that activate HER receptors, except HER-2, are expressed as trans-membrane precursors and contain a conserved structural region, the EGF-like domain. This family consists of 13 members, each of which binds a specific receptor and induce the homo- or hetero-dimerization of HER receptors [13, 14]. EGF, transforming growth factor alpha (TGF*α*), betacellulin (BC), amphiregulin (AR), epiregulin (EPR), heparin-binding EGF-like ligand (HB-EGF) and epigen are HER-1 ligands. Neuregulin (also known as heregulin) 1 and 2 (NRG1, NRG2 or HRG1, HRG2) are HER-3 and HER-4 ligands, while HER-4 has additionally NRG3, NRG4 and share ligands BC, EPR, HB-EGF and epigen with HER-1 receptor. HER-2 is a “ligand-free” receptor and can activate its TK domain through auto-phosphorylation after homo- or hetero-dimerization with other HER partners.

Based on HER-2 over-expression at protein level or gene amplification, breast cancer patients are treated with anti-HER-2 agents. Herceptin (trastuzumab) is an antibody against the extracellular domain of HER-2 receptor [6]. Pertuzumab, another novel anti-HER-2 antibody binds to the dimerization sites of the HER-2 receptor inhibiting more effectively its dimerization and HRG-induced activation through HER-3 [15]. Combinational therapy of HER-2 breast carcinomas with trastuzumab and pertuzumab has shown positive clinical results [16, 17]. Additionally, HER-2 / HER-1 TK inhibitors, such as lapatinib, are being used in breast cancer therapeutics [18]. So far it was believed that activation of HER-2 occurs only when HER-2 is over-expressed, amplified or in the presence of a truncated form. Many breast cancer patients present intrinsic or acquired resistance, attributed to various mechanisms, to anti-HER-2-directed therapies [19]. Growing evidence support the participation of HER-3 and HRGs in activation of HER-2, regardless of its expression. Preliminary results show that HER-2 over-expression might not be necessary and activation of HER-3 and/or HER-4 by HRGs might be enough to subsequently activate HER-2 [20–23]. There is a notion that patients with increased levels of HRGs but negative or low expression of HER-2 and low or high HER-3 expression, could benefit from treatment with anti-HER-2 agents [24].

Most protein biomarker studies, regarding HER family, have focused on blood and tissue samples, examining solely one member of this family. The ability to detect specific protein–protein interactions and post-translational modifications in blood and/or tissue as biomarkers for a plethora of human pathological conditions holds great promise for molecular medicine. The proximity ligation assay (PLA) is a versatile molecular tool that has many advantages, such as minimal sample consumption and flexible assay reconfiguration [25]. In the present study we used PLA to study the dimerization pattern of HER protein family members after treatment of MCF-7 (not HER-2 amplified) and SKBR3 (HER-2 amplified) human breast cancer cells with or without HRG1 and various HER-2 / HER-3 blocking agents (trastuzumab, pertuzumab and U3-1287). In addition, the HER dimerization pattern was evaluated in paraffin-embedded human breast cancer tissues with various HER-2 expression levels.

## RESULTS

### HER dimerization pattern after the addition of heregulin

**Figure 1:**
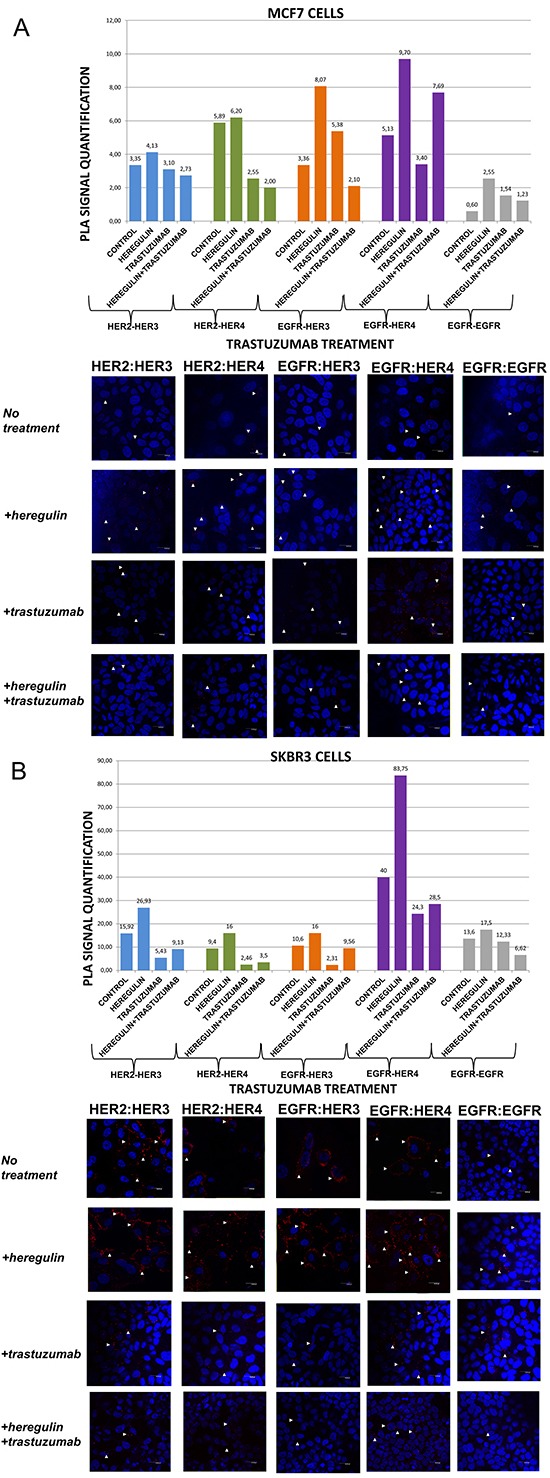
Dimer formation pattern in MCF-7 cells (A) and SKBR3 cells The data analysis was done using Duolink Image Tool Software and representative images of each context are shown. White arrowheads point to positive signal (red dots) of dimer formation using the Duolink *in situ* PLA (scale bar, 20 μm). EGFR = HER-1; PLA, proximity ligation assay. **Dimer formation pattern in MCF-7 cells (B) after the addition of heregulin (HRG) and trastuzumab.** The data analysis was done using Duolink Image Tool Software and representative images of each context are shown. White arrowheads point to positive signal (red dots) of dimer formation using the Duolink *in situ* PLA (scale bar, 20 μm). EGFR = HER-1; PLA, proximity ligation assay.

**Figure 2:**
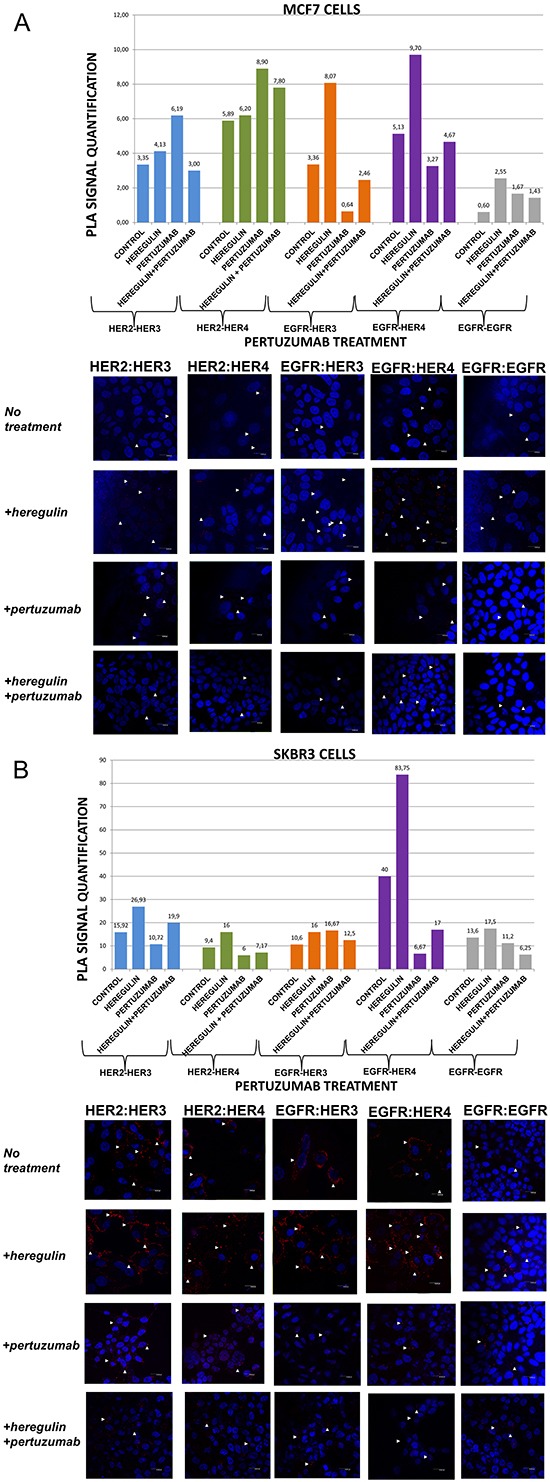
Dimer formation pattern in MCF-7 cells (A) and SKBR3 cells The data analysis was done using Duolink Image Tool Software and representative images of each context are shown. White arrowheads point to positive signal (red dots) of dimer formation using the Duolink *in situ* PLA (scale bar, 20 μm). EGFR = HER-1; PLA, proximity ligation assay. **Dimer formation pattern in MCF-7 cells (B) after the addition of heregulin (HRG) and pertuzumab.** The data analysis was done using Duolink Image Tool Software and representative images of each context are shown. White arrowheads point to positive signal (red dots) of dimer formation using the Duolink *in situ* PLA (scale bar, 20 μm). EGFR = HER-1; PLA, proximity ligation assay.

**Figure 3:**
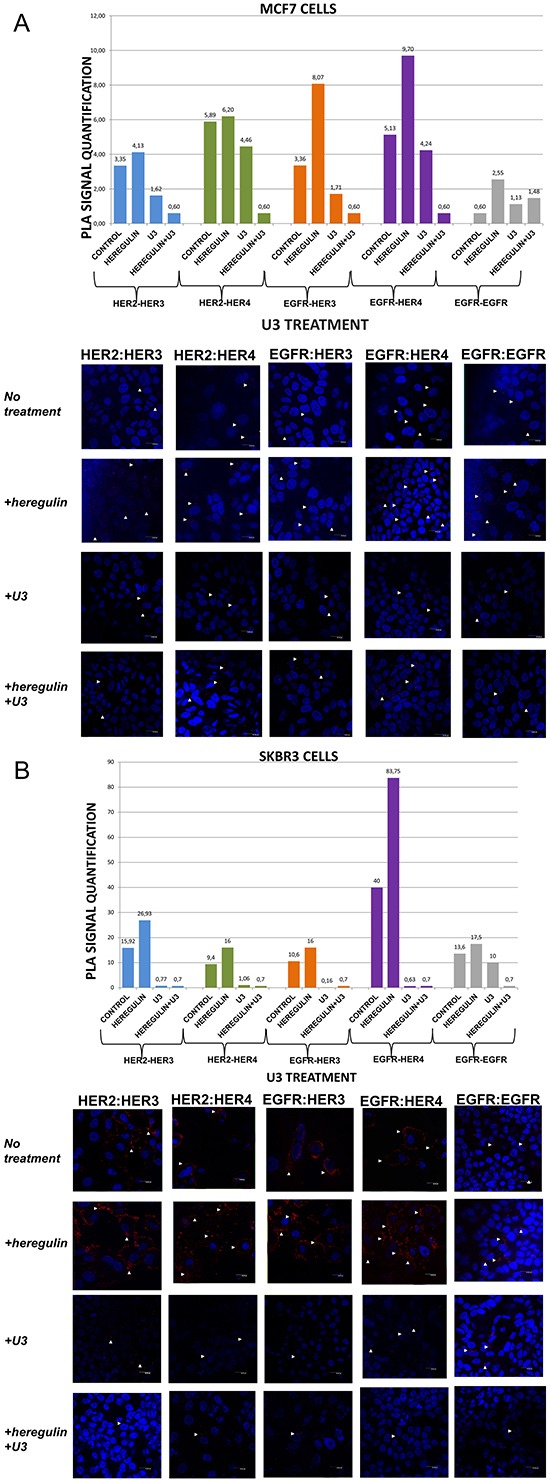
Dimer formation pattern in MCF-7 cells (A) and SKBR3 cells The data analysis was done using Duolink Image Tool Software and representative images of each context are shown. White arrowheads point to positive signal (red dots) of dimer formation using the Duolink *in situ* PLA (scale bar, 20 μm). EGFR = HER-1; PLA, proximity ligation assay. **Dimer formation pattern in MCF-7 cells (B) after the addition of heregulin (HRG) and U3.** The data analysis was done using Duolink Image Tool Software and representative images of each context are shown. White arrowheads point to positive signal (red dots) of dimer formation using the Duolink *in situ* PLA (scale bar, 20 μm). EGFR = HER-1; PLA, proximity ligation assay.

**Figure 4:**
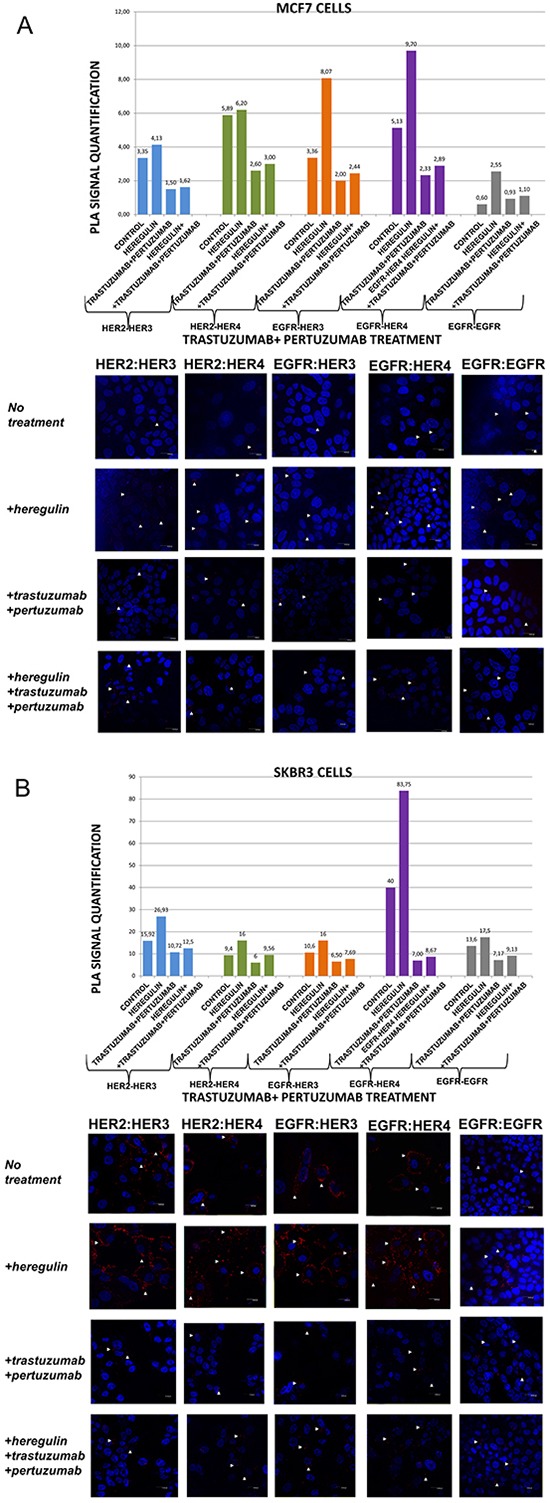
Dimer formation pattern in MCF-7 cells (A) and SKBR3 cells The data analysis was done using Duolink Image Tool Software and representative images of each context are shown. White arrowheads point to positive signal (red dots) of dimer formation using the Duolink *in situ* PLA (scale bar, 20 μm). EGFR = HER-1; PLA, proximity ligation assay. **Dimer formation pattern in MCF-7 cells (B) after the addition of heregulin (HRG) and trastuzumab plus pertuzumab.** The data analysis was done using Duolink Image Tool Software and representative images of each context are shown. White arrowheads point to positive signal (red dots) of dimer formation using the Duolink *in situ* PLA (scale bar, 20 μm). EGFR = HER-1; PLA, proximity ligation assay.

**Figure 5:**
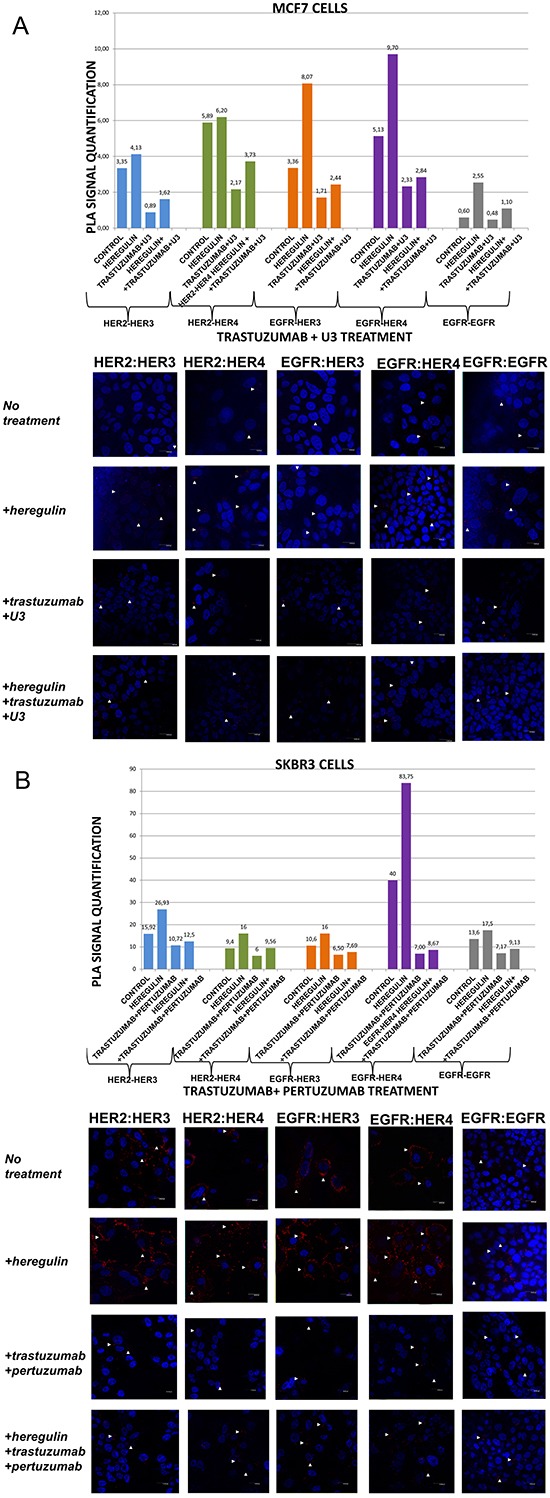
Dimer formation pattern in MCF-7 cells (A) and SKBR3 cells The data analysis was done using Duolink Image Tool Software and representative images of each context are shown. White arrowheads point to positive signal (red dots) of dimer formation using the Duolink *in situ* PLA (scale bar, 20 μm). EGFR = HER-1; PLA, proximity ligation assay. **Dimer formation pattern in MCF-7 cells (B) after the addition of heregulin (HRG) and trastuzumab plus U3.** The data analysis was done using Duolink Image Tool Software and representative images of each context are shown. White arrowheads point to positive signal (red dots) of dimer formation using the Duolink *in situ* PLA (scale bar, 20 μm). EGFR = HER-1; PLA, proximity ligation assay.

We initially investigated the formation of HER dimers with and without the addition of heregulin (HRG) (Figures 1–5).

In MCF-7 cells (PLA signal scales 0-12), there was a high incidence of HER-1:HER-3 (8,07 *vs* 3,36) and HER-1:HER-4 (9,70 *vs* 5,13) heterodimers as well as HER-1:HER-1 (2,55 *vs* 0,60) homodimer formation. As expected, subtle differences were observed after the addition of HRG regarding formation of HER-2:HER-3 and HER-2:HER-4 heterodimers. HRG is the main ligand that activates HER-3 and HER-4 [23]. In MCF-7 breast cancer cell line, HRG has been shown to increase a sustained mitogen-activated protein kinase (MAPK) signal activity favoring differentiation, whereas EGF only elicits a transient MAPK signal activity favoring cell proliferation [26, 27]. The identification of the dimerization pattern as well as the transcriptional networks stimulated by HER receptors under different conditions is important for better understanding breast cancer progression.

Contrary to MCF-7 cells, in SKBR3 cells (PLA signal scales 0-90) there was a high incidence of HER-1:HER-4 (83,75 *vs* 40) and HER-2:HER-3 (26,93 *vs* 15,92) heterodimers, while there was only a small amount of HER-1:HER-1 (17,5 *vs* 13,6) homodimers. Furthermore, a slight increase of HER-1:HER-3 (16 *vs* 10,6) and HER-2:HER-4 (16 *vs* 9,4) heterodimers was also found. It seems that in HER-2 positive breast cancer cells, at least *in vitro*, HRG stimulates the formation of certain HER-3- and HER-4-based heterodimers, thus generating new data regarding the way we could therapeutically target them.

### HER dimerization pattern after the addition of trastuzumab

We next examined the formation of HER dimers with and without the addition of HRG and trastuzumab (T) (Figure 1A and 1B). T is a humanized monoclonal antibody that binds to domain IV of the extracellular portion of HER-2 receptor and has generated significant clinical results in the treatment of early stage and metastatic HER-2 positive breast cancer patients [6].

In MCF-7 cells (PLA signal scales 0-12), the co-treatment of cells with HRG and T compared to T alone resulted in a lower percentage of HER-1:HER-3 (2,10 *vs* 5,38) and a higher percentage of HER-1:HER-4 (7,69 *vs* 3,40) heterodimer formation. Notably, there was a reduction in HER-2:HER-4 heterodimer formation after treatment with T compared with the addition of HRG alone, while no discernible difference was observed after the combination of HRG with T *vs* T alone. Subtle differences were detected after the combination of T and HRG regarding the formation of HER-2:HER-3 heterodimer and HER-1:HER-1 homodimer (Figure 1A).

In SKBR3 cells (PLA signal scales 0-90), the co-treatment of cells with HRG and T compared to T alone resulted in a higher percentage of HER-1:HER-3 (9,56 *vs* 2,31) and a slight higher percentage of HER-1:HER-4 (28,5 *vs* 24,3) heterodimer formation. Of note, a reduction in HER-2:HER-4 and HER-2:HER-3 heterodimer formation after treatment with T was found compared with the addition of HRG alone, albeit an increase was observed after the combination of HRG with T *vs* T alone. Notable differences were also detected after the combination of T and HRG compared to HRG alone regarding the formation of HER-1:HER-1 homodimer (6,62 *vs* 17,5), suggesting that in HER-2 positive breast cancer cells, at least *in vitro*, HRG might also act as HER-1 activating ligand (Figure 1B).

### HER dimerization pattern after the addition of pertuzumab

We went on to explore the formation of HER dimers with and without the addition of HRG and pertuzumab (P) (Figure 2A and 2B). P is a monoclonal antibody that binds to domain II of the extracellular portion of HER-2 receptor and inhibits ligand-mediated dimerization of HER-2 with other protein family members, among them HER-3 [28]. P has been recently approved for the treatment of metastatic and locally advanced (as neoadjuvant treatment) HER-2 positive breast cancer patients in combination with T and chemotherapy [16, 17].

In MCF-7 cells (PLA signal scales 0-12), the co-treatment of cells with HRG and P compared to P alone resulted, contrarily to the addition of T, in a higher percentage of HER-1:HER-3 (2,46 *vs* 0,64) as well as HER-1:HER-4 (4,67 *vs* 3,27) heterodimer formation. Noteworthy, there was an increase in HER-2:HER-4 heterodimer formation after treatment with P, opposite to the addition of T, compared to the addition of HRG alone, while no discernible difference was found after the combination of HRG with P *vs* P alone. Notable reduction of HER-2:HER-3 heterodimer (3,0 *vs* 6,19) was detected after the combination of P and HRG, contrarily to the combination of T and HRG, while P increased HER-2:HER-3 heterodimer formation relevant to HRG alone. Additionally, no discernible difference was observed regarding the formation of HER-1:HER-1 homodimer (Figure 2A).

In SKBR3 cells (PLA signal scales 0-90), the co-treatment of cells with HRG and P compared to P alone, opposite to the addition of T, resulted in a considerably higher percentage of HER-1:HER-4 (17 *vs* 6,67) and a slightly lower percentage of HER-1:HER-3 (12,5 *vs* 16,67) heterodimer formation. Notably, a reduction in HER-2:HER-4 and HER-2:HER-3 heterodimer formation after treatment with P was found compared with the addition of HRG alone, although an increase was detected after the combination of HRG with P *vs* P alone. Discernible differences were also detected after the combination of P and HRG compared to HRG alone regarding the formation of HER-1:HER-1 homodimer (6,25 *vs* 17,5) (Figure 2B).

### HER dimerization pattern after the addition of U3-1287 (U3)

We then examined the formation of HER dimers with and without the addition of HRG and U3-1287 (Figure 3A and 3B). U3-1287 (U3), now known as AMG 888, is a HER-3 fully humanized neutralizing monoclonal antibody that has been previously shown to decrease HER-3 from the cell surface and also inhibit the augmentation of HER-3 following HER-2 inhibition. Moreover, U3 in combination with HER-2 inhibitors can activate apoptosis *in vitro*, partially restore sensitivity to T in T-resistant xenografts and improves their survival [29]. U3 has been also reported to inhibit HER-3-induced extracellular signal-regulated kinase (ERK) signaling and *in vitro* and *in vivo* growth of multiple tumor cell lines as single agent or in combination with other HER inhibitors [30, 31]. Although the exact biochemical properties of U3 have not been disclosed yet, it is currently in early clinical testing [32, 33]. Nevertheless, no clinical data are so far available regarding its activity in breast carcinomas, while predictive biomarkers that are being evaluated are primarily based on HRG levels and not on HER-3 dimerization pattern [34].

In MCF-7 cells (PLA signal scales 0-12), the co-treatment of cells with HRG and U3 compared to U3 alone resulted in a lower percentage of HER-1:HER-3 (0,60 *vs* 1,71) and HER-1:HER-4 (0,60 *vs* 4,24) heterodimer formation. Of note, there was also a reduction in HER-2:HER-4 heterodimer formation compared with the addition of HRG or U3 alone. Notable reduction of HER-2:HER-3 heterodimer (0,60 *vs* 1,62) was detected after the combination of U3 and HRG, while subtle differences were observed with the same combination regarding the formation of HER-1:HER-1 homodimer (Figure 3A). Therefore, it seems that U3, through its direct anti-HER-3 activity, exhibits more potent inhibition of HER dimerization compared to T and P after treatment with HRG.

In SKBR3 cells (PLA signal scales 0-90), the co-treatment of cells with HRG and U3 compared to HRG alone resulted in an impressively low percentage of HER-1:HER-4 (0,7 *vs* 83,75) and HER-1:HER-3 (0,7 *vs* 16) heterodimer formation, while no difference was detected with the combination of HRG plus U3 *vs* U3 alone. Remarkably, there was also a discernible reduction in HER-2:HER-4 (0,7 *vs* 16) and HER-2:HER-3 (0,7 *vs* 26,93) heterodimer formation after the addition of U3 compared with the addition of HRG alone, while again no difference was detected with the combination of HRG plus U3 *vs* U3 alone. Notable differences were also detected after the combination of U3 and HRG compared to HRG alone regarding the formation of HER-1:HER-1 homodimer (0,7 *vs* 17,5) (Figure 3B).

These results drive to the following assumptions: i) U3 has more potent activity than T and P in suppressing dimer formation in HER-2 positive breast cancer cells; ii) HRG addition does not seem to correlate with U3 dimerization-inhibiting activity in HER-2 positive breast cancer cells and might not represent a valuable predictive biomarker of its activity; iii) in HER-2 positive breast cancer cells, at least *in vitro*, HRG might act as HER-1-activating ligand and U3 seems to inhibit this activity.

### HER dimerization pattern after the combination of trastuzumab and pertuzumab

We subsequently investigated the formation of HER dimers with and without the addition of HRG and T plus P combination (Figure 4A and 4B).

In MCF-7 cells (PLA signal scales 0-12), the co-treatment of cells with HRG and T plus P compared to T plus P alone resulted in similar results with the combination of HRG and P alone. Noteworthy, there was a reduction in HER-2:HER-4 (3 *vs* 6,2) and HER-2:HER-3 (1,62 *vs* 4,13) heterodimer formation compared with the addition of HRG alone, while no discernible difference was found after the combination of HRG with T plus P *vs* T plus P alone. Subtle difference was also detected with the same combination regarding the formation of HER-1:HER-1 homodimer (Figure 4A).

In SKBR3 cells (PLA signal scales 0-90), the co-treatment of cells with HRG and T plus P compared to T plus P alone resulted in a bit higher percentage of HER-1:HER-4 and HER-1:HER-3 heterodimer formation. Of note, there was also a reduction in HER-2:HER-4 (9,56 *vs* 16) and HER-2:HER-3 (12,5 *vs* 26,93) heterodimer formation compared with the addition of HRG alone, whereas a slight increase was found after the combination of HRG with T plus P *vs* T plus P alone. Notable differences were also detected after the combination of T plus P and HRG compared with HRG alone regarding the formation of HER-1:HER-1 homodimer (9,13 *vs* 17,5) (Figure 4B).

### HER dimerization pattern after the combination of trastuzumab and U3-1287

We finally explored the formation of HER dimers with and without the addition of HRG and T plus U3 (Figure 5A and 5B).

In MCF-7 cells (PLA signal scales 0-12), the co-treatment of cells with HRG and T plus U3 compared to T plus U3 alone resulted in a slightly higher percentage of HER-1:HER-3 (2,44 *vs* 1,71) and HER-1:HER-4 (2,84 *vs* 2,33) heterodimer formation. Notably, there was also an increase of HER-2:HER-4 and HER-2:HER-3 heterodimers as well as HER-1:HER-1 homodimer compared with the addition of T plus U3 alone (Figure 5A). These results imply that the addition of T possibly partly suppresses the activity of U3 against HRG-induced dimer formation.

In SKBR3 cells (PLA signal scales 0-90), the co-treatment of cells with HRG and T plus U3 compared to HRG alone resulted again in a strikingly low percentage of HER-1:HER-4 (8,77 *vs* 83,75) and HER-1:HER-3 (2,44 *vs* 16) heterodimer formation, while no difference was detected with the combination of HRG with T plus U3 *vs* T plus U3 alone. Interestingly enough, there was also a discernible reduction in HER-2:HER-4 (3,82 *vs* 16) and HER-2:HER-3 (2,81 *vs* 26,93) heterodimer formation after the addition of T plus U3 compared with the addition of HRG alone, while again no difference was detected with the combination of HRG and T plus U3 *vs* T plus U3 alone. Notable differences were also observed after the combination of T plus U3 and HRG compared to HRG alone regarding the formation of HER-1:HER-1 homodimer (1,27 *vs* 17,5) (Figure 5B).

These data in conjunction with those presented earlier put in the assumption that adding T to U3 in HER-2 positive breast cancer cells does not increase the potential of U3 to inhibit HER dimerization.

### HER dimerization pattern in human breast tumor tissues

**Figure 6:**
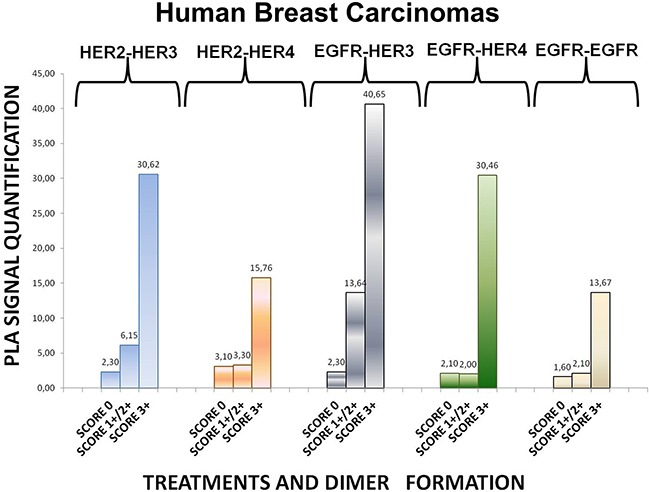
Dimer formation pattern in three groups of human breast cancer tissues based on HER-2 expression profile The data analysis was done using Duolink Image Tool Software. EGFR = HER-1; PLA, proximity ligation assay.

**Figure 7:**
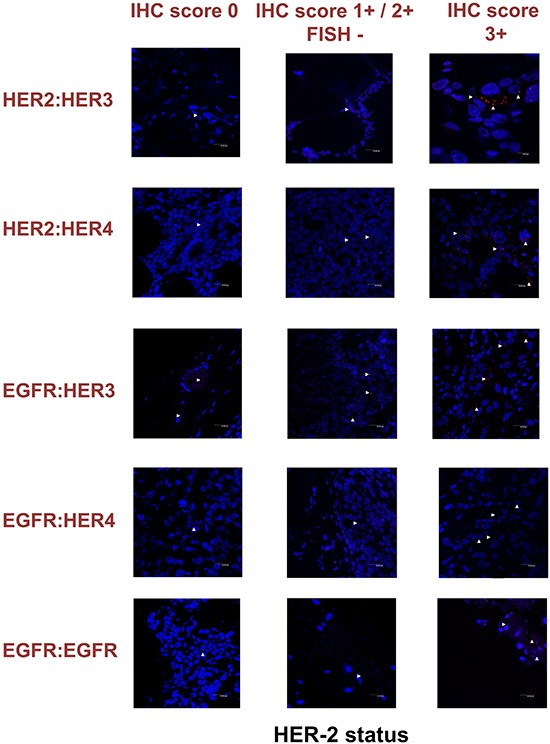
Dimer formation pattern in three groups of human breast cancer tissues based on HER-2 expression profile is shown in representative fluorescence images White arrowheads point to positive signal (red dots) of dimer formation using the Duolink *in situ* PLA (scale bar, 20 μm). EGFR = HER-1; IHC, immunohistochemistry; FISH, fluorescence *in situ* hybridization.

To expand our findings in the *in vivo* setting, we employed PLA to examine the dimerization pattern of HER protein family members in human paraffin-embedded breast cancer tissues with three HER-2 expression patterns, based on the HER-2 evaluation recommendation guidelines of ASCO/CAP [35]. To this end we assessed 5 breast tumor samples with HER-2 immunohistochemistry (IHC) score 0 (group 1), 5 breast tumor samples with HER-2 IHC score 1+ / 2+ and fluorescence *in situ* hybridization (FISH) negative (group 2) and 5 breast tumor samples with HER-2 IHC score 3+ and FISH positive (group 3). As anticipated, in group 3 a high frequency of membrane-bound HER-2:HER-3 and HER-1:HER-3 heterodimers was detected, whilst a high number of HER-1:HER-4 heterodimer and HER-1:HER-1 homodimer was also found (Figures 6 and 7). This is in concert with the results obtained in SKBR3 cells (Figures 1B, 2B, 3B, 4B, 5B). Therefore, in group 3 patients the application of HER-2 and/or HER-3 targeting agents seems the most rational therapeutic strategy. The role of HER-4 in breast carcinogenesis is still obscure but the increased percentage of HER-1:HER-4 heterodimers in this group of patients might represent a separate redundant mechanism by which HER-4 stimulates EGFR signaling events and thus consists a dismal dimerization profile [36]. The formation of HER-1:HER-1 homodimers seems to be in harmony with the previously described theory of pre-existing homodimers that may alter receptor–ligand binding properties [37], while it verifies the notion that HER-2 holds a central role regarding HER-1 and HER-3 levels as well as their dimerization patterns [38]. However, as described above, HER-1:HER-4 heterodimer and HER-1:HER-1 homodimer can be effectively decreased after treatment with HER-2 and/or HER-3 targeting agents (Figures 1B, 2B, 3B, 4B, 5B).

Remarkably, in group 2 patients among all evaluated HER dimers, the most prevalent was the HER-1:HER-3 heterodimer, which probably represents a percentage of HER-2 negative breast tumors that may bear the potential to show growth inhibition and hence clinical benefit with HER-3-directed agents (Figures 6 and 7). This is partly in agreement with the results obtained in MCF-7 cells (Figures 1A, 2A, 3A, 4A, 5A). Though the expression of HRG which has been used as a promising predictive biomarker in recent studies with HER-3 inhibitors [39] was not measured, based on our *in vitro* data we pose that PLA-detected dimerization pattern is a more reliable and easily reproducible predictive biomarker for HER-3 targeting agents, since HRG is a ligand for both HER-3 and HER-4, and probably HER-1 (Figures 1A, 2A, 3A, 4A, 5A). In group 1 patients no HER dimer was detected in significant amounts, in accordance with the existing knowledge that in such tumors HER targeting has no biological rationale and does not offer any clinical benefit (Figures 6 and 7).

## DISCUSSION

Over-expression and/or increased activation of HER receptors have been demonstrated in breast carcinomas. These receptors usually exist as inactive monomers, with the exception of HER-2. Their dimerization is an essential step for their stimulation and downstream activity. Exactly which dimers are assembled each time is dependent on the available ligands and their relative affinities for each receptor. Following ligand binding the two receptor-monomers associate and the TK domain is activated through trans-phosphorylation. Dynamic technologies have evaluated the significance of HER expression and downstream pathways on transcriptional regulation [38, 40]. However, HER dimerization pattern is context-dependent and correlates with the downstream effective networks. HER dimers have been investigated with co-immunoprecipitation and immunohistochemical methods [41]. In the present study we employed *in situ* PLA to identify membrane-bound protein–protein interactions of HER receptors at various conditions [42]. To date, the data regarding the evaluation of HER dimerization pattern with PLA are scarce [43–46]. For example, PLA has been previously used for the detection of HER-2:HER-3 heterodimers in human tissues and this detection has been correlated with dismal clinical outcome [46]. However, herein we examined, for the first time, the dimerization pattern of all HER family members after the addition of HRG1 and HER-2 / HER-3 inhibitors (trastuzumab, pertuzumab and U3). Additionally, we assessed HER dimerization pattern in human breast cancer tissues that were categorized in three groups according to their HER-2 expression level.

HRG ligands are vitally engaged in normal mammary gland development and function as well as in breast carcinogenesis. The HRG family comprises four genes and their differentially spliced mRNA products give rise to several variant proteins. HRG1 is a ligand for HER-3 and HER-4 receptors and activates HER-2 through the dimerization process with them, as we also found in MCF-7 and SKBR3 cells. HRG1a, 2a, 2b display the highest expression in breast cancers compared with other HER ligands and have been correlated with worst overall survival [47]. It has been shown that HRG1 acts mainly through HER-3 receptor and may stimulate the acquisition of cancer stem cell-like characteristics [48]. Additionally, it has been reported that HRG1 (broadly expressed in the brain), through the formation of HER-2:HER-3 heterodimers, increases proliferation potential and breast cancer cell migration capacity via the brain microvascular endothelia [49]. HRG can also activate anchorage-independent breast cancer cell growth more potently than EGF, whilst the HRG-dependent activation of phosphatidylinositol-3-kinase (PI3K)/Akt pathway is a necessary event for cell transformation [50]. Ligand specificity dictates the combinatorial formation of certain HER dimer species, which activate intracellular pathways in a dimer-specific manner. In this vein, we unexpectedly found that in SKBR3 (HER-2 amplified) cells HRG1 might also act as HER-1 activating ligand, highlighting the complexity and plasticity of HER dimerization process. Various computational models have been generated in order to elucidate HER signaling events during breast cancer evolution [51, 52], although a definitive one is still missing.

The presence of HRG ligands in breast cancer tissues is thought to represent a potential resistance mechanism to anti-HER-2 targeting agents, such as trastuzumab and pertuzumab, as they can activate remnant-non-bound HER-2 receptors through HER-3 binding [14, 53, 54]. Autocrine or paracrine-derived HRG1 can also activate the formation of HER-2:HER-3 heterodimers, which has been found to be effectively blocked by pertuzumab, but not trastuzumab [55]. In order to clarify the effect of HRG1 and HER-3 on HER dimerization, we evaluated in breast cancer cell lines the dimer formation pattern after the addition of trastuzumab, pertuzumab and their combination with or without HRG1. Our results are in accord with previous reports regarding HER-3-based dimers. However, we found that HER-1- and HER-4-based dimer formation is differently stimulated by trastuzumab and pertuzumab and that their combinatorial use produces greater reduction of all HER-1- and HER-4-based dimers, thus generating a better clinical effect in breast cancer cells.

HER-3 is usually co-expressed with other receptor TKs in breast cancer cells [1]. It promotes tumor initiation and progression through activation of the PI3K/Akt pathway, which participates in breast cancer progression as well as in resistance mechanism of currently used endocrine and anti-HER-2 targeted regimens [56]. HER-3 targeting with ATP-competitive small molecules is not feasible, since this receptor lacks intrinsic TK activity. Nevertheless, new HER-3 selective inhibitors are being created in order to inhibit its dimerization capacity with HER-1 and/or HER-2 [57, 58]. Furthermore, various monoclonal antibodies targeting HER-3 are being developed and evaluated regarding their ability to degrade the receptor and suppress downstream signaling [59, 60]. Importantly, some of these antibodies have been engineered in such a way to also target other membrane receptors (e.g. HER-1, HER-2, insulin-like growth factor 1 receptor (IGF-1R)) [61]. Several of these compounds have already entered clinical testing, while potential biomarkers of HER-3 targeting agents are being investigated [9, 18]. In this vein, we evaluated in breast cancer cell lines the dimer formation pattern after the addition of trastuzumab, U3 and their combination with or without HRG1. Our data reveal that HER-3 selective inhibitors have more potent activity regarding HER dimerization inhibition, compared with trastuzumab and/or pertuzumab. Additionally, we found that the combination of trastuzumab and U3 might not have an additive inhibition in receptor dimerization of HER-2 positive breast cancer cells, and may have competitive action in HRG-induced receptor dimerization, raising concerns regarding the ongoing clinical testing of this combination (NCT01512199). Another important finding of our study was that the presence of HRG1 did not suppress U3 inhibitory capacity in HER-2 positive breast cancer cells. All these *in vitro* results combined with the dimerization pattern in HER-2 positive human breast cancer tissues suggest that HRG1 expression is not a suitable predictive factor for HER-3 targeting agents, while the formation of HER-1:HER-4 heterodimer may represent a potential predictive factor for this class of agents. Moreover, our *in vitro* and *in vivo* results identified a breast cancer sub-group (HER-2 IHC 1+ / 2+ and FISH negative with high frequency of HER-1:HER-3 heterodimer) that might benefit from HER-3 selective targeting agents, irrespectively of HRG1 expression. Nonetheless, it should be noted that the above are only hypotheses that need to be tested.

A potential drawback of our study relies on the issue that although HER receptors are membrane proteins there is growing evidence of nuclear translocation and function [62], while no cell viability, apoptosis and/or signaling assay were performed in conjunction with PLA after the various treatments in order to correlate the dimerization effects with these assessments *in vitro*. Full-length nuclear HER-1 is correlated with transcriptional regulation, DNA replication and DNA repair. In several tumors HER-1 has been detected in the nucleus of cancer cells and these patients have a remarkably poor outcome [63]. HER-3 has been also found in the nucleus of human mammary epithelial cells, while HRG1 stimulation can shift HER-3 from the nucleolus to the nucleus and then to the cytoplasm [64]. In addition, it has been demonstrated that HRG1-activated nuclear HER-4 receptor stimulates the aggressive behavior of breast tumor cells [65], which is partially in agreement with our results regarding the role of HER-4-based dimers. Therefore, HER expression and dimerization pattern in breast carcinomas should be further investigated in conjunction with their subcellular localization.

## MATERIALS AND METHODS

### Cell lines and cell culture

MCF-7 and SKBR3 human breast adenocarcinoma cell lines were purchased from ATCC Bioresource Center in 2009 and 2001, respectively. The cell lines were authenticated prior to experimentation at the Laboratory of Genetics of the Biomedical Research Foundation of the Academy of Athens (Athens, Greece) by inverted DAPI banding karyotyping method. DMEM media, L-glutamine, fetal bovine serum (FBS) and antibiotics were purchased from Life Technologies. Both cell lines were grown in DMEM supplemented with 10% FBS, 2 mmol/L glutamine and antibiotics (100 U/ml penicillin-streptomycin) in a humidified atmosphere of 5% C0_2_ at 37°C.

### Reagents

The basic reagents used in our experiments and their sources are: HRG1-*β*1 10 μM (R&D Systems), pertuzumab (P) 30 μM (Genentech), U3 inhibitor 30 μM (U3 Pharma) and trastuzumab (T) 30 μM (Roche). Primary antibodies anti-EGFR (HER-1) mAb (ab30), anti-EGFR (HER-1) mAb (103575) and anti-HER-3 mAb ([2F9] 91084) were purchased from Abcam (Abcam, USA). Anti-HER-2 ([H-200] 134481) and anti-HER-4 ([L-20] 31149) mAbs were obtained from Santa Cruz (SCBT, USA). The DUOLINK In Situ Ligation Kit was purchased from Olink (DUO92002-Duolink In Situ PLA Probe Anti-Rabbit PLUS, DUO92004 - Duolink In Situ PLA Probe Anti-Mouse MINUS, DUO92006 - Duolink In Situ PLA Probe Anti-Goat MINUS, DUO92007 - Duolink In Situ Detection Reagents Orange) (Olink Bioscience, Uppsala, Sweden). Nuclei were stained with TOPRO3 (T3605) from Life Technologies (Thermo Fisher Scientific).

### Proximity ligation assay (PLA)

Proximity ligation assay (PLA) is a method that retains the dependency of proximal binding of antibodies and provides a mean for signal amplification [66]. This technology utilizes DNA oligonucleotides conjugated to antibodies (proximity probes) to provide a template for ligation of subsequently added circularization DNA oligonucleotides. The ligation will only generate circular products if the proximity probes bind in close proximity. The length and orientation of the oligonucleotides, as well as the size of the affinity reagents, determine the distance requirement for *in situ* PLA. Once a circular ligation product is formed, it can be amplified by rolling circle amplification (RCA). The signal amplification provided by RCA facilitates detection of single ligation events [42]. In general, *in situ* PLA can be used for visualization of endogenous protein–protein interactions [66], post-translational modifications [67] as well as protein–nucleic acid interactions [68, 69], while the generation of discrete signals facilitates enumeration and analysis by a specific digital image processing tool. The ability to determine protein interactions in paraffin-fixed tissue sections may facilitate the use of these techniques in every day clinical practice. PLA probes anti-goat PLUS, anti-mouse MINUS and detection reagent DUOLINK II orange were obtained from Olink Bioscience.

### PLA on cell lines

MCF-7 and SKBR3 cells were cultured on glass coverslips. After reaching ∼80% confluence in culture, cells were starved overnight and then treated for 48 h. Subsequently, the cells were fixed with 4% PFA in PBS for 20 min at room temperature. After permeabilization for 10 min at room temperature, PLA was performed as described in the Duolink II protocol using different sets of primary antibodies (anti-HER-2/HER-3, anti-HER-2/HER-4, anti-EGFR (HER-1)/HER-3, anti-EGFR (HER-1)/HER-4 and anti-EGFR (HER-1)/EGFR (HER-1)). For controls, one of the primary antibodies of each set was omitted and the protocol was carried out without any further changes. Cells were then incubated with the PLA probes diluted 1:5 in antibody diluent (Olink Bioscience) in a humidified chamber for 1 h at 37°C. Subsequent hybridization, ligation, amplification and detection were performed as per manufacturer's instructions. Before mounting the glass coverslips on slides, cells were incubated for 2 min with TOPRO3 (1:1000 in PBS) for nuclei staining. Finally, coverslips were mounted with MOWIOL mounting medium (Sigma Aldrich). Fluorescence images were acquired using a Zeiss Axiovert microscope (Carl Zeiss Microscopy, Thornwood, NY USA). For each coverslip, images were taken from at least 10 randomly selected fields of view. Data analysis was performed using Duolink Image Tool Software that has been developed for quantification of PLA signals (Olink Bioscience) [70]. For statistical analysis, the resulting “PLA signals per cell” in all groups were normalized to the mean value of the control group. Representative images for each condition were prepared for figure presentation by applying brightness and contrast adjustments uniformly using Adobe Photoshop CS5.

### PLA on human tissue samples

The study included patients diagnosed with breast carcinoma at the Department of Propaedeutic Surgery, University of Athens Medical School, ‘Laikon’ General Hospital; cancer tissue samples were retrieved from the archive of the First Department of Pathology, University of Athens Medical School. For the paraffin-embedded human tissue samples (PFETs), three groups of samples were tested based on HER-2 IHC expression and FISH score (IHC score 0, IHC score 1+ / 2+, FISH negative and IHC score 3+, FISH positive). Each group included 5 different samples and experiments were repeated three times. The PFETs were de-paraffinized following a standard protocol of xylene and ethanol incubations, antigen retrieval (1 mM Tris-EDTA, pH 9.0), wash with ddH_2_0 and PBS and all other reactions (blocking, primary antibodies, PLA probes, ligation, amplification and cover slips mounting) according to manufacturer's instructions. Fluorescence images were acquired using a Zeiss Axiovert microscope. For each coverslip, images were taken from at least 10 randomly selected fields of view. Data analysis was done using Duolink Image Tool Software [70].

### Ethics statement

All experiments were carried out in accordance with the guidelines and regulations set by the Ethics Committee of the Medical School of National and Kapodistrian University of Athens. Furthermore, the entire study protocol was approved by the Ethics Committee of the Medical School of National and Kapodistrian University of Athens and follows the principles of the Declaration of Helsinki. Informed consent was obtained from all patients.

### Data analysis

All experiments were performed at least three times and data analysis was done using Duolink Image Tool Software (Olink Bioscience) [70]. For further analysis, the resulting “PLA signals per cell” in all groups were normalized to the mean value of the control group. Representative images for each condition were prepared for figure presentation by applying brightness and contrast adjustments uniformly using Adobe Photoshop CS5.

## Abbreviations

AR: amphiregulin
BC: betacellulin
EGFR: epidermal growth factor receptor
EMT: epithelial–mesenchymal transition
EPR: epiregulin
ERK: extracellular signal-regulated kinase
FBS: fetal bovine serum
FISH: fluorescence *in situ* hybridization
HB-EGF: heparin-binding EGF-like ligand
HRG: heregulin
IGF-1R: insulin-like growth factor 1 receptor
IHC: immunohistochemistry
MAPK: mitogen-activated protein kinase
NRG: neuregulin
P: pertuzumab
PFETs: paraffin-embedded human tissue samples
PI3K: phosphatidylinositol-3-kinase
PLA: proximity ligation assay
T: trastuzumab
TGF*α*: transforming growth factor alpha
TK: tyrosine kinase
